# The ability of long non-coding RNA RP11-284N8.3 to predict the risk, the severity and 28-day mortality of adults with sepsis

**DOI:** 10.1097/MD.0000000000033355

**Published:** 2023-03-24

**Authors:** Yanwei Cheng, Ning Ding, Xue Cao, Jiaoyang Wang, Jiange Zhang, Xiaopeng Shi, Lijun Xu, Lijie Qin

**Affiliations:** a Department of Emergency, Henan Provincial People’s Hospital, People’s Hospital of Zhengzhou University, People’s Hospital of Henan University, Zhengzhou, China; b Department of Rheumatology and Immunology, Henan Provincial People’s Hospital, People’s Hospital of Zhengzhou University, People’s Hospital of Henan University, Zhengzhou, China.

**Keywords:** 28-day mortality, lncRNA RP11-284N8.3, predictive value, sepsis, sepsis severity

## Abstract

In a prior study, we identified a novel sepsis specific long noncoding RNAs (lncRNA) RP11-284N8.3, which may primarily participate in T cell activation and immune response during sepsis. However, the clinical significance of lncRNA RP11-284N8.3 in sepsis remains entirely unknown. This single-center prospective cohort study enrolled 147 adults with sepsis and 74 healthy controls (HCs) with matched age and sex between January 2021 and November 2022 at our hospital. Blood samples and clinical data were collected from HCs at enrollment and from adults with sepsis within 24 hours after admission. lncRNA RP11-284N8.3 expression was detected by RT-qPCR. The relative expression of lncRNA RP11-284N8.3 was significantly decreased in adults with sepsis compared to HCs (*P* < .0001), in adults with septic shock compared to adults without shock (*P* = .0012), and in 28-day deaths compared to 28-day survivors (*P* = .0006). receiver operating characteristic curves of lncRNA RP11-284N8.3 in predicting sepsis severity and 28-day mortality showed an area under the curve of 0.6570 (95% confidence interval [CI]: 0.5701–0.7440) and an area under the curve of 0.6765 (95% CI: 0.5809–0.7721), respectively. Multivariate logistic regression analysis revealed that lncRNA RP11-284N8.3 was an independent risk factor for 28-day mortality in adults with sepsis (odds ratio: 0.1057, 95% CI: 0.0115–0.7746, *P* = .0328). Low expression of lncRNA RP11-284N8.3 is correlated with increased risk, severity and 28-day mortality in adults with sepsis, and it may function as a potential biomarker to facilitate the diagnosis and management of sepsis.

## 1. Introduction

Sepsis is defined as a life-threatening multiorgan dysfunction caused by a dysregulated host response to infection.^[1]^ Annually, approximately 48.9 million population are affected by sepsis, over 11 million of those affected die, and one 6th of sepsis survivors experience significant functional limitations.^[2–4]^ In view of the high morbidity, disability and mortality, sepsis has now been established as the quintessential medical disorder of the twenty-first century.^[5]^ Recent decades have seen the remarkable progress of treatments for sepsis, including fluid therapy, vasoactive drug, antimicrobial agent, etc. Unfortunately sepsis remains the most common cause of death in the intensive care unit (ICU) due to delayed diagnosis and inadequate management in the initial hours. Under such a background, an urgent investigation into novel biomarkers contributing to identify the risk of sepsis timely and monitor the prognosis of sepsis precisely would be significant.

Over the past decade, long noncoding RNAs (lncRNAs) have become an active area of sepsis research. lncRNAs are a group of nonprotein-coding transcripts with more than 200 nucleotides in length^[6]^ and have been reported to exert key regulatory roles in the pathogenesis of various diseases.^[7–11]^ With the advance of high throughput sequencing technology, an increasing number of sepsis-associated lncRNAs had been identified.^[12–16]^ In our prior study,^[17]^ the lncRNA-mRNA network in sepsis was constructed based on a publicly available RNA-seq data, and we identified a potential key sepsis-associated lncRNA RP11-284N8.3. At present, lncRNA RP11-284N8.3 is largely with unknown functions in sepsis. However, we found most mRNAs highly co-expressed with lncRNA RP11-284N8.3 primarily enriched in immune response and T cell activation related Gene Ontology biological process terms. Our results indicated that the novel sepsis specific lncRNA RP11-284N8.3 may exert potential roles in regulating immune response during sepsis and may be closely related to the development and progression of sepsis. Nonetheless, the clinical significance of lncRNA RP11-284N8.3 in sepsis patients remains largely unknown.

In the present study, we aim to investigate the expression of lncRNA RP11-284N8.3 from peripheral blood monouclear cells (PBMCs) with risk, severity and 28-day mortality in adults with sepsis. The findings may contribute to the understanding of lncRNA RP11-284N8.3 in sepsis and provid a new insight into sepsis management.

## 2. Materials and Methods

### 2.1. Study population

This was a single-center prospective cohort study performed in Henan Provincial People’s Hospital. A total of 173 sepsis patients were admitted to our emergency intensive care unit within the initial 24 hours from January 2021 to November 2022. According to the “sepsis-3” consensus,^[1]^ all patients were diagnosed with sepsis. Patients were excluded if they were aged under 18 years or over 80 years, transferred from another hospital, receiving immunosuppressive therapy, concomitant with preexisting autoimmune diseases, suffering from malignancies, or in pregnancy or lactation. Within that same time frame, 73 healthy individuals with matched sex and age features were chosen as healthy controls (HCs), who all underwent physical examination in our hospital.

The present study was approved by the ethics committee of Henan Provincial People’s Hospital (2021-49). The written informed consent was obtained from all participant or their guardians.

### 2.2. Clinical data collection

The clinical data of all enrolled adults with sepsis were obtained from clinical electronic medical records, including demographics (age, gender, and smoke), chronic comorbidities, laboratory data measured at admission (white blood cell, blood platelet, C-reactive protein [CRP], procalcitonin [PCT], total bilirubin, serum creatinine, albumin, serum electrolyte, arterial lactic acid), and primary infection site, disease severity (SOFA score, APACHE II score, septic shock) assessed by a senior doctor. Patients were followed up until death or 28 days after ICU admission, and 28-day mortality was calculated as well. Based on the survival status in 28-day follow up, sepsis patients were further classified as 28-day survivors and 28-day deaths. In addition, the demographics and laboratory data of HCs were also recorded at enrollment.

### 2.3. Sample collection and qRT-PCR detection

Peripheral blood samples were collected from HCs at enrollment and from sepsis patients within 24 hours after admission. All the blood samples were processed immediately after collection for the isolation of PBMCs, which were stored at − 80°C before RNA extraction. Total RNA was extracted from PBMCs using the TRIzol reagent (Invitrogen) according to the manufacturer’s instructions. Then, the purified RNA was reverse-transcribed taken for complementary DNA by PrimeScript RT reagent Kit (Takara). Subsequently, qRT-PCR was conducted by using TB Green Fast qPCR Mix (Takara) and specific primers (forward: 5′-GTCCTCCACTAATCACAGAAT-3′, reverse: 5′-TCACTTGATGTCAGAATGCT-3′). Relative gene expression was determined by employing the 2^−ΔΔCT^ method and normalized against GAPDH.

### 2.4. Statistical analysis

All statistical analyses were carried out using GraphPad Prism 9.0 software (GraphPad Software lnc., San Diego, California). Continuous variables were presented as mean ± standard deviation or median and inter-quarter range, as appropriate, depending on the normality of the data, which was assessed using Kolmogorov-Smirnov test. Normally distributed continuous variables were compared using Student *t* test, while the continuous variables that were not normally distributed were compared using Mann–Whitney *U* test. Categorical variables were present as counts and proportions, and were tested using Chi-square test or Fisher exact test. Correlation analysis was determined by Spearman rank correlation test. The receiver operating characteristic (ROC) curve analysis was used to determine the predictive accuracy of lncRNA RP11-284N8.3 in predicting the risk, severity and 28-day mortality of sepsis, and the area under the curve (AUC) (odds ratio [OR], 95% confidence interval [CI]) was calculated. Logistic regression analysis was used to determine 28-day mortality-related factors. After univariate analysis, variables with *P* value < .10 were included in multivariate regression analysis. A 2-sided *P* value < .05 was considered statistically significant.

## 3. Results

### 3.1. Baseline and clinical characteristics of HCs and adults with sepsis

A total of 173 adults with sepsis admitted to our emergency intensive care unit within the initial 24 hours between January 2021 and November 2022 were consecutively enrolled. Of whom, 26 adults were excluded as their family members quitted aggressive treatment or rescue for economic reasons. Finally, 147 adults with sepsis and 73 HCs were analyzed in this study.

Baseline and clinical characteristics of HCs and adults with sepsis can be viewed in Table 1. The median age of sepsis patients was 62 (49, 71) years, and there were 87 (59.18%) males. Thirty (20.41%) patients had a history of smoking. No difference of age (*P* = .2277), gender (*P* = .3148) and history of smoking (*P* = .4805) between sepsis patients and HCs was observed. Forty-three (29.25%) had a chronic comorbidity of hypertension, and 55 (37.41%) had a primary respiratory infection. Regarding laboratory results at admission, increased levels of white blood cell (*P* < .0001), CRP (*P* < .0001), PCT (*P* < .0001) and serum creatinine (*P* < .0001), while decreased levels of blood platelet (*P* = .0287) and albumin (*P* = .0003) were observed in sepsis patients compared to HCs. The mean lactic acid level in sepsis patients was 1.64 ± 1.09 mmol/L. The levels of total bilirubin (*P* = .6147), sodium (*P* = .8363), potassium (*P* = .8869), and calcium (*P* = .6993) did not differ significantly between sepsis patients and HCs. Regarding sepsis severity score, APACHE II score and SOFA score showed a mean value of 18.77 ± 3.08 and 10.71 ± 2.28, respectively.

**Table 1:** Baseline and clinical characteristics of the study population.

Items	Sepsis patients (n = 147)	HCs (n = 73)	*P* value
Demographics			
Age, yr, median (IQR)	62 (49, 71)	59 (46.5, 69)	.2277
Male, n (%)	87 (59.18)	38 (52.05)	.3148
Smoke, n (%)	30 (20.41)	12 (16.44)	.4805
Chronic comorbidities,			
Hypertension, n (%)	43 (29.25)	-	-
Diabetes, n (%)	29 (19.73)	-	-
Heart disease, n (%)	23 (15.65)	-	-
Respiratory disease, n (%)	26 (17.69)	-	-
Hepatic insufficiency, n (%)	4 (2.72)	-	-
Renal insufficiency, n (%)	15 (10.20)	-	-
Cerebrovascular disease, n (%)	21 (14.29)	-	-
Laboratory tests			
WBC, 10^9^/L, median (IQR)	16.30 (9.40, 16.30)	6.93 (4.55, 7.10)	<.0001
PLT, 10^9^/L, median (IQR)	154 (80, 245)	199 (177, 269)	.0287
CRP, mg/L, median (IQR)	80.60 (29.47, 106.33)	5.54 (3.32, 7.51)	<.0001
PCT, ng/mL, median (IQR)	4.21 (2.03, 8.10)	0.15 (0.08, 0.20)	<.0001
Albumin, g/L, median (IQR)	31.5 (25.5, 35.9)	44.3 (41.5, 47.2)	.0003
Tbil, umol/L, median (IQR)	12.8 (8.3, 22.5)	11.8 (6.7, 13.8)	.6147
Scr, umol/L, median (IQR)	104 (92, 133)	61 (44, 70)	<.0001
Sodium, mmol/L, median (IQR)	140.0 (135.0, 145.0)	142.0 (138.0, 146.0)	.8363
Potassium, mmol/L, median (IQR)	4.0 (3.6, 4.8)	4.1, (3.8, 4.9)	.8869
Calcium, mmol/L, mean ± SD	2.05 ± 0.23	2.07 ± 0.21	.6993
Lactic acid, mmol/L, mean ± SD	1.64 ± 1.09	-	-
Primary infection site			
Respiratory infection, n (%)	55 (37.41)	-	-
Abdominal infection, n (%)	33 (22.45)	-	-
Blood stream infection, n (%)	18 (12.24)	-	-
Urinary infection, n (%)	9 (6.12)	-	-
Other infections, n (%)	32 (21.77)	-	-
Disease severity score			
APACHE II score, mean ± SD	18.77 ± 3.08	-	-
SOFA score, mean ± SD	10.71 ± 2.28	-	-

CRP = C-reactive protein, HCs = healthy controls, IQR = inter-quarter range, PCT = procalcitonin, PLT = blood platelet, SD = standard deviation, Scr = serum creatinine, TBil = total bilirubin, WBC = white blood cell.

### 3.2. Relative expression of lncRNA RP11-284N8.3 in HCs and adults with sepsis

**Figure 1.**
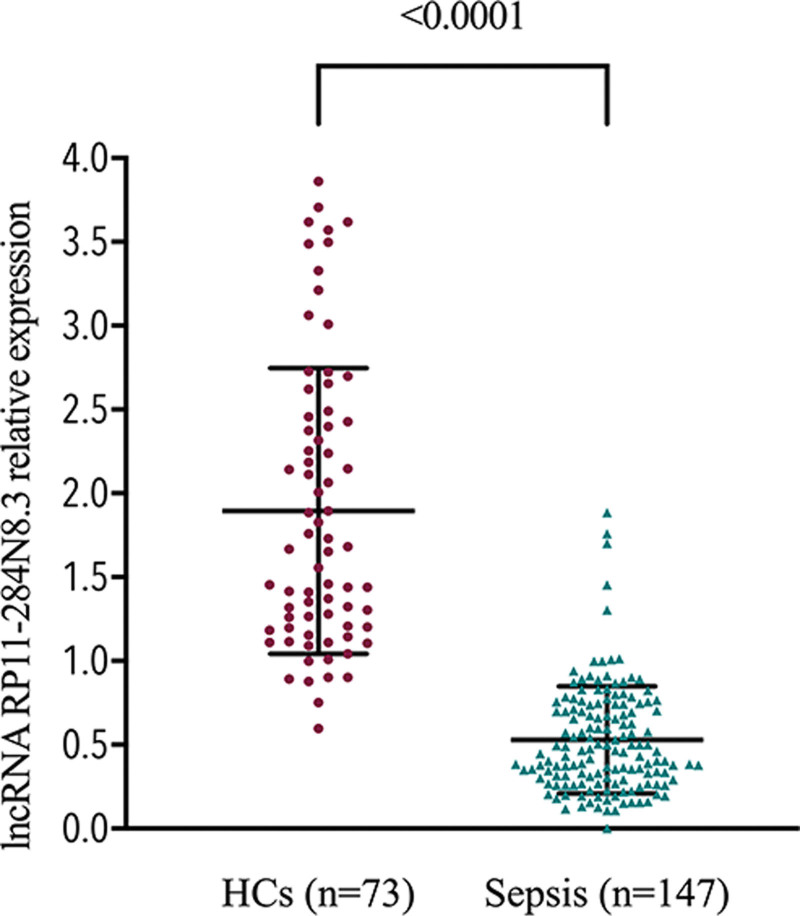
The relative expression of lncRNA RP11-284N8.3 was significantly decreased in patients with sepsis compared to HCs. HCs = healthy controls.

The relative expression of lncRNA RP11-284N8.3 from PBMCs was significantly decreased in adults with sepsis than in HCs (median: 0.4644 [0.2939, 0.7448]) vs 1.670 [1.203, 2.441], *P* < .0001) (Fig. 1). This result indicated that lncRNA RP11-284N8.3 were closely correlated with sepsis risk.

### 3.3. Predictive value of lncRNA RP11-284N8.3 for sepsis severity

**Figure 2.**
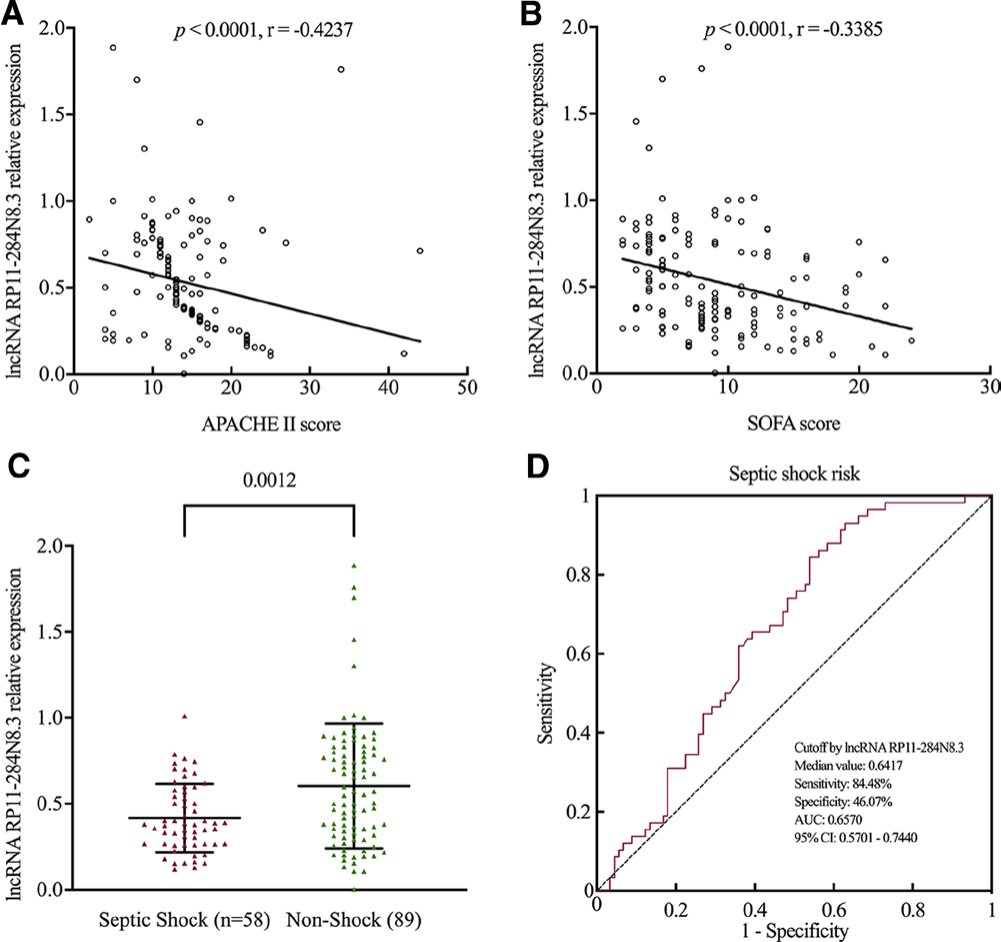
The predictive value of lncRNA RP11-284N8.3 for sepsis severity. (A) The relative expression of lncRNA RP11-284N8.3 was negatively correlated with APACHE II score (r = −0.4237, *P* < .0001) in sepsis patients. (B) The relative expression of lncRNA RP11-284N8.3 was negatively correlated with SOFA score (r = −0.3385, *P* < .0001) in sepsis patients. (C) The relative expression of lncRNA RP11-284N8.3 showed decreased in patients with septic shock compared to non-shock patients. (D) ROC curve of lncRNA RP11-284N8.3 in predicting sepsis shock showed an AUC of 0.6570 (95% CI: 0.5701–0.7440, *P* < .0001). AUC = area under the curve, CI = confidence interval, ROC = receiver operating characteristic.

All enrolled adults with sepsis were evaluated by a senior doctor for disease severity, including APACHE II score, SOFA score, septic shock or non-shock. The relative expression of lncRNA RP11-284N8.3 was negatively correlated with APACHE II score (r = −0.4237, *P* < .0001) (Fig. 2A) and SOFA score (r = −0.3385, *P* < .0001) (Fig. 2B) in adults with sepsis. In addition, the relative expression of lncRNA RP11-284N8.3 showed a decrease in adults with septic shock compared to that in non-shock adults, (median: 0.3810 [0.2668, 0.5733] vs 0.5525 [0.3234, 0.8163], *P* = .0012) (Fig. 2C). ROC curve showed moderate predictive value for lncRNA RP11-284N8.3 in distinguishing adults with septic shock from those without septic shock, with an AUC of 0.6570 (95% CI: 0.5701–0.7440, *P* < .0001) and a cutoff value of 0.6417 (sensitivity: 84.48%, specificity: 46.07%) (Fig. 2D). These results indicated that lncRNA RP11-284N8.3 from PBMCs was negatively correlated with disease severity in adults with sepsis.

### 3.4. LncRNA RP11-284N8.3 is an independent risk factor for 28-day mortality in adults with sepsis

**Figure 3.**
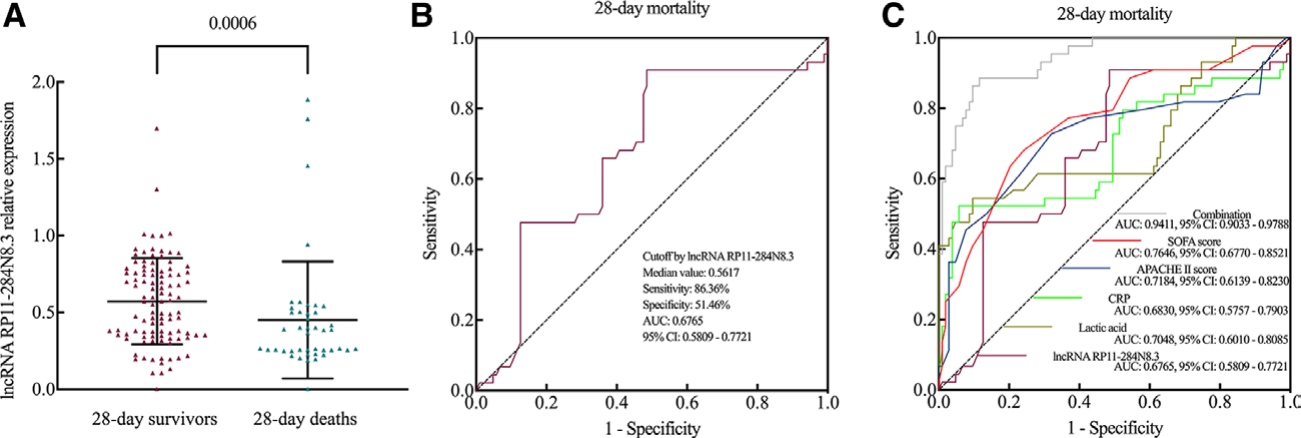
The predictive value of lncRNA RP11-284N8.3 for 28-day mortality in sepsis patients. (A) The relative expression of lncRNA RP11-284N8.3 was decreased in deaths compared to survivors. (B) ROC curve of lncRNA RP11-284N8.3 in predicting *28-day mortality in sepsis patients* showed an AUC of 0.6765 (95% CI: 0.5809–0.7721, *P* = .0007). (C) ROC curve showed that the combination of lncRNA RP11-284N8.3, CRP, lactic acid, APACHE II score and SOFA score had an AUC of 0.9411 (95% CI: 0.9033–0.9788, *P* < .0001). AUC = area under the curve, CI = confidence interval, CRP = C-reactive protein, ROC = receiver operating characteristic.

All adults with sepsis were divided into 28-day survivors (n = 103) and 28-day deaths (n = 44) according to the survival status in 28-day follow up. The detailed information of demographics and clinical characteristics of survivors and deaths groups is shown in Table 2. The relative expression of lncRNA RP11-284N8.3 was decreased in deaths compared to survivors (median: 0.3772 [0.2578, 0.5043] vs 0.5735 [0.3546, 0.7688], *P* = .0006) (Table 2 and Fig. 3A). After relevant confounders adjustment using multivariate regression model, lncRNA RP11-284N8.3 (OR: 0.1057, 95% CI: 0.0115–0.7746, *P* = .0328) was an independent factor for 28-day mortality in adults with sepsis (Table 2). Other independent factors including age (OR: 1.055, 95% CI: 1.008–1.115, *P* = .0337), CRP (OR: 1.027, 95% CI: 1.013–1.045, *P* = .0007), PCT (OR: 1.18, 95% CI: 1.073–1.327, *P* = .0019), lactic acid (OR: 1.736, 95% CI: 0.9841–3.383, *P* = .0745), APACHE II score (OR: 1.235, 95% CI: 1.087–1.453, *P* = .0045) and SOFA score (OR: 1.348, 95% CI: 1.183–1.582, *P* < .0001) were also associated with 28-day mortality (Table 3).

**Table 2:** Baseline, clinical characteristics and lncRNA RP11-284N8.3 relative expression in survivors and deaths groups.

Items	28-day survivors (n = 103)	28-day deaths (n = 44)	*P* value
Demographics			
Age, yr, median (IQR)	59 (46, 70)	66.5 (54, 72.75)	.0300
Male, n (%)	63 (61.17)	24 (54.55)	.4546
Smoke, n (%)	19 (18.45)	11 (25.00)	.4805
Chronic comorbidities,			
Hypertension, n (%)	28 (27.18)	15 (34.88)	.4261
Diabetes, n (%)	17 (16.50)	12 (27.27)	.1739
Heart disease, n (%)	14 (13.59)	9 (20.45)	.3256
Respiratory disease, n (%)	20 (19.42)	6 (13.64)	.4842
Hepatic insufficiency, n (%)	3 (2.91)	1 (2.27)	>.9999
Renal insufficiency, n (%)	9 (8.65)	6 (13.64)	.3798
Cerebrovascular disease, n (%)	12 (11.65)	9 (20.45)	.1991
Laboratory tests			
WBC, 10^9^/L, median (IQR)	12.50 (10.00, 15.50)	13.96 (7.55, 21.40)	.6437
PLT, 10^9^/L, median (IQR)	134 (93, 252)	127 (86, 247)	.7581
CRP, mg/L, median (IQR)	58.5 (27.9, 97.6)	104.6 (54.8, 158.2)	.0004
PCT, ng/mL, median (IQR)	4.29 (2.21, 7.60)	4.35 (1.75, 14.75)	.6543
Albumin, g/L, median (IQR)	30.9. (28.1, 37.6)	29.7 (28.4, 33.4)	.5011
Tbil, umol/L, median (IQR)	12.0 (8.7, 23.5)	14.6 (11.7, 26.4)	.2274
Scr, umol/L, median (IQR)	101 (97, 107)	108 (80, 174)	.1655
Sodium, mmol/L, median (IQR)	140.0 (135.0, 145.0)	140.0 (135.0, 145.0)	.7040
Potassium, mmol/L, median (IQR)	4.3 (3.8, 5.2)	4.1 (3.6, 4.8)	.5860
Calcium, mmol/L, mean ± SD	2.1 ± 0.1	2.1 ± 0.0	.9830
Lactic acid, mmol/L, mean ± SD	1.42 ± 0.88	2.15 ± 1.36	.0001
Primary infection site			
Respiratory infection, n (%)	39 (37.86)	16 (36.36)	>.9999
Abdominal infection, n (%)	21 (20.39)	12 (27.27)	.3916
Blood stream infection, n (%)	13 (12.62)	5 (11.36)	>.9999
Urinary infection, n (%)	4 (3.88)	5 (11.36)	.1276
Other infections, n (%)	26 (25.24)	6 (13.64)	.1326
Disease severity score			
APACHE II score, mean ± SD	13.01 ± 4.02	17.68 ± 2.23	<.0001
SOFA score, mean ± SD	7.71 ± 3.99	12.59 ± 4.56	<.0001
lncRNA RP11-284N8.3	0.5735 (0.3546, 0.7688)	0.3772 (0.2578, 0.5043)	.0006

CRP = C-reactive protein, IQR = inter-quarter range, PCT = procalcitonin, PLT = blood platelet, SD = standard deviation, Scr = serum creatinine, TBil = total bilirubin, WBC = white blood cell.

**Table 3:** Logistic regression models of factors related to 28-day mortality in sepsis patients.

	Univariate regression model				Multivariate regression model		
Items	OR	95% CI	*P* value	OR		95% CI	*P* value
Age	1.031	1.005–1.060	.0251	1.055		1.008–1.115	.0337
CRP	1.017	1.009–1.025	<.0001	1.027		1.013–1.045	.0007
PCT	1.068	1.024–1.131	.0094	1.18		1.073–1.327	.0019
Scr	0.9921	0.9850–0.9974	.0122	0.9909		0.9797–0.9985	.0593
Lactic acid	1.95	1.364–2.918	.0005	1.736		0.9841–3.383	.0745
Urinary infection	3.173	0.8003–13.41	.0976	1.942		0.2215–17.28	.5464
APACHE II score	1.17	1.084–1.279	.0002	1.235		1.087–1.453	.0045
SOFA score	1.237	1.140–1.357	<.0001	1.348		1.183–1.582	<.0001
lncRNA RP11-284N8.3	0.2311	0.0535–0.8331	.0361	0.1057		0.0115–0.7746	.0328

CI = confidence interval, CRP = C-reactive protein, OR = odds ratio, PCT = procalcitonin, Scr = serum creatinine.

### 3.5. Predictive value of lncRNA RP11-284N8.3 for 28-day mortality in adults with sepsis

ROC curve revealed that the AUC of lncRNA RP11-284N8.3 in predicting 28-day mortality risk was 0.6765 (95% CI: 0.5809–0.7721, *P* = .0007) (Fig. 3B). Using a cutoff value of 0.5617 for predicting 28-day mortality risk, the sensitivity was 86.36%, and the specificity was 51.46%. In addition, SOFA score (AUC: 0.7646, 95% CI: 0.6770–0.8521, *P* < .0001), APACHE II score (AUC: 0.7184, 95% CI: 0.6139–0.8230, *P* < .0001), lactic acid (AUC: 0.7048, 95% CI: 0.6010–0.8085, *P* < .0001), and CRP (AUC: 0.6830, 95% CI: 0.5757–0.7903, *P* = .0005) all had predictive value on 28-day mortality risk. The combination of these aforementioned factors (AUC: 0.9411, 95% CI: 0.9033–0.9788, *P* < .0001) showed strong predictive value on 28-day mortality risk in adults with sepsis (Fig. 3C).

## 4. Discussion

lncRNAs have been demonstrated to exert key regulatory roles in all sorts of biological processes, thereby contributing to the development and progression of diverse diseases, including sepsis.^[18,19]^ In our prior study,^[17]^ we profiled the differentially expressed lncRNAs and mRNAs co-expression network in sepsis based on a publicly available RNA-seq data, and identified 3 sepsis specific lncRNAs, including RP11-284N8.3, CTB-61M7.2, and LINC00861. Among of them, lncRNA RP11-284N8.3 is entirely unknown to sepsis and may primarily participate in T cell activation and immune response based on the functional enrichment analysis of its highly co-expressed mRNAs. As we all know, sepsis initiates a complicated immunopathogenesis process that involves both innate and adaptive immune responses.^[20,21]^ Thus, there is no doubt that lncRNA RP11-284N8.3 is associated with the occurrence and development of sepsis.

Prior studies have revealed that lncRNAs may function as potential biomarkers for the diagnosis and prognosis of sepsis, such as the well-known lncRNAs NEAT1 and MALAT1.^[14,22,23]^ Herein, we discovered that the relative expression of lncRNA RP11-284N8.3 was remarkably decreased in adults with sepsis compared to that in HCs, which suggested that lncRNA RP11-284N8.3 were closely correlated with sepsis. Additionally, we explored the correlation of lncRNA RP11-284N8.3 with disease severity in adults with sepsis. It is well-known that APACHE II score and SOFA score have become the most accepted and used among the general ICU severity of illness scoring systems.^[24]^ We found that lncRNA RP11-284N8.3 was negatively correlated with APACHE II score and SOFA score. Septic shock is the most severe form of sepsis and is a state of circulatory failure that occurs in a subset of sepsis patients.^[25]^ Lower expression of lncRNA RP11-284N8.3 were also observed in adults with septic shock compared to adults without septic shock. Taken together, lncRNA RP11-284N8.3 can serve as a novel biomarker for predicting sepsis risk and severity.

Besides, we also found that the relative expression lncRNA RP11-284N8.3 was decreased in 28-day deaths compared to 28-day survivors, and it was an independent factor with a moderate degree of predictive value for 28-day mortality risk in adults with sepsis (AUC: 0.6765, 95% CI: 0.5809–0.7721). The value was numerically similar to that of CRP and lactic acid, but was weaker than that of APACHE II score and SOFA score. Notably, ROC curve showed that the combination of lncRNA RP11-284N8.3, CRP, lactic acid, APACHE II score and SOFA score could well-predict 28-day mortality risk in adults with sepsis, suggesting that it might be a potential tool to recognize sepsis patients who have a high 28-day mortality risk, which may improve the management toward these patients.

Despite the interesting results, the limitations of this study are as follows: First, the relatively small sample size might result in poor statistical power. Future studies with larger sample sizes should be conducted. Second, our single-center study might cause selected bias. Future studies of large scale and multi-center are needed. Third, the expression level of lncRNA RP11-284N8.3 was detected only once for each patient, while dynamic detection of lncRNA RP11-284N8.3 may yield more persuasive results. Finally, it is unclear how lncRNA RP11-284N8.3 affects sepsis and regulates the exact molecular signaling pathways. Future studies are required to investigate the detailed mechanism of lncRNA RP11-284N8.3 underlying sepsis.

## 5. Conclusions

To the best of our knowledge, this is the first study to evaluate the predictive value of lncRNA RP11-284N8.3 for risk, severity and 28-day mortality in adults with sepsis. Our results demonstrated that lncRNA RP11-284N8.3 was down-regulated in sepsis patients and was negatively correlated with sepsis severity and possessed a moderate predictive value on 28-day mortality risk. LncRNA RP11-284N8.3 could serve as a potential biomarker for facilitating diagnosis and management in sepsis patients.

## Author contributions

**Conceptualization:** Yanwei Cheng, Lijie Qin.

**Data curation:** Ning Ding.

**Software:** Xue Cao, Jiange Zhang.

**Investigation:** Jiaoyang Wang.

**Validation:** Xiaopeng Shi.

**Writing – original draft:** Yanwei Cheng.

**Writing – review & editing:** Lijun Xu.
